# TRIM32: A Multifunctional Protein Involved in Muscle Homeostasis, Glucose Metabolism, and Tumorigenesis

**DOI:** 10.3390/biom11030408

**Published:** 2021-03-10

**Authors:** Simranjot Bawa, Rosanna Piccirillo, Erika R. Geisbrecht

**Affiliations:** 1 Department of Biochemistry and Molecular Biophysics, Kansas State University, Manhattan, KS 66506, USA; simranbawa@ksu.edu; 2 Department of Neuroscience, Istituto di Ricerche Farmacologiche Mario Negri IRCCS, 20156 Milan, Italy; rosanna.piccirillo@marionegri.it

**Keywords:** muscle, costamere, muscular dystrophy, cancer

## Abstract

Human tripartite motif family of proteins 32 (TRIM32) is a ubiquitous multifunctional protein that has demonstrated roles in differentiation, muscle physiology and regeneration, and tumor suppression. Mutations in TRIM32 result in two clinically diverse diseases. A mutation in the B-box domain gives rise to Bardet–Biedl syndrome (BBS), a disease whose clinical presentation shares no muscle pathology, while mutations in the NHL (NCL-1, HT2A, LIN-41) repeats of TRIM32 causes limb-girdle muscular dystrophy type 2H (LGMD2H). TRIM32 also functions as a tumor suppressor, but paradoxically is overexpressed in certain types of cancer. Recent evidence supports a role for TRIM32 in glycolytic-mediated cell growth, thus providing a possible mechanism for TRIM32 in the accumulation of cellular biomass during regeneration and tumorigenesis, including in vitro and in vivo approaches, to understand the broad spectrum of TRIM32 functions. A special emphasis is placed on the utility of the *Drosophila* model, a unique system to study glycolysis and anabolic pathways that contribute to the growth and homeostasis of both normal and tumor tissues.

## 1. The TRIM Family of Proteins

**Figure 1:**
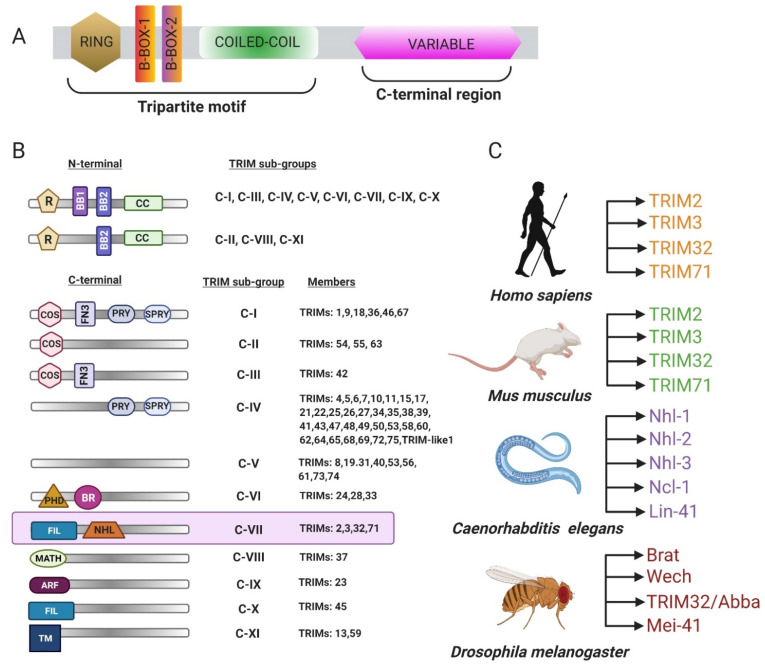
Classification of the tripartite motif family of proteins 32 (TRIM). (**A**) General schematic of a TRIM protein. The N-terminus is comprised of a really interesting new gene (RING) domain, one or two B-box regions, and a coiled-coil domain followed by a variable C-terminus. (**B**) Table representing different categories of TRIM proteins. (**C**) Different NHL (NCL-1, HT2A, LIN-41) proteins encoded in vertebrates and invertebrates (adapted from WILLIAMS et al. 2019).

The tripartite motif family of proteins (TRIM) is characterized by the presence of an N-terminal RING (really interesting new gene) finger followed by one or two B-box domains (BB1 and BB2) and a coiled-coil region with a variable C-terminus (Figure 1A) [1,2]. The TRIM family consists of approximately 70 protein members involved in a plethora of biological processes, including apoptosis, cell cycle regulation, muscle homeostasis, and the innate immune response [3,4]. Each domain has independent functions, and the variability in the C-terminal region defines the structural and biochemical properties of the protein and imparts target specificity. The TRIM family of proteins is sorted into nine categories classified as C-I through C-XI based upon the composition of the C-terminal domain (Figure 1B) [2]. The C-VII subclass includes proteins with five or six NHL (NCL-1, HT2A, LIN-41) repeats, which are thought to primarily mediate protein–protein interactions. TRIM-NHL proteins also bind RNAs and are key regulators of cell growth, proliferation, and differentiation [5]. There are four TRIM-NHL proteins encoded in *Drosophila melanogaster*, *Mus musculus*, and *Homo sapiens* followed by five in *Caenorhabditis elegans* [5] (Figure 1C).

One of these TRIM-NHL proteins, TRIM32, was uncovered as a protein that binds to HIV-1 Tat, a key transactivator of viral transcription [6]. Since this initial discovery, the biological roles of TRIM32 have rapidly expanded [2,3,7,8,9]. Human TRIM32 is expressed in a variety of tissues, including skeletal muscle, with elevated levels eminent in the brain and heart [10]. The *Drosophila* ortholog of TRIM32 is enriched in larval and adult muscle tissue and demonstrates structural and functional conservation across species [11,12,13]. This review highlights classical and emerging roles of TRIM32 as a multifunctional protein in a multitude of developmental and physiological functions, as well as summarizes its involvement in regulating glycolytic enzymes to promote growth in both normal and cancerous tissues.

### 1.1. Structural and Functional Organization of TRIM32

**Figure 2:**
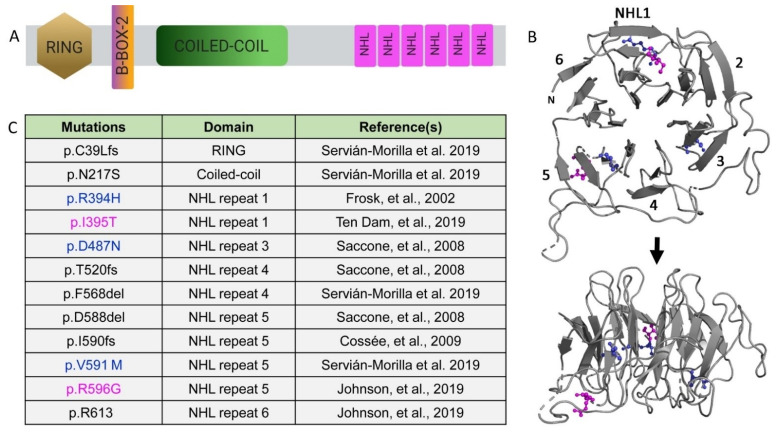
Limb-girdle muscular dystrophy type 2H (LGMD2H)-causing TRIM32 mutations in the NHL domains. (**A**) Schematic of the TRIM32 protein. (**B**) Ribbon diagram showing the face (top panel) and 90° rotation (bottom panel) of human NHL domains. Colored amino acids are listed in panel B. (**C**) Table illustrating mutations causative for LGMD2H. Known point mutations are in color, with blue representing amino acids strictly conserved in the *Drosophila* TRIM32 sequence.

Mammalian TRIM32 is a part of the largest subfamily of RING E3 ligases involved in regulating multifaceted post-translational modifications of cellular proteins through ubiquitination [2,7]. The N-terminus consists of the typical TRIM motifs, including the catalytic RING domain, a single B-box domain (Type II), and a coiled-coil region, followed by six C-terminal NHL repeats (Figure 2A). Conserved cysteine and histidine residues spaced out in the core of the RING domain coordinate two zinc ions in a cross-bridge fashion to provide structural maintenance and to ensure biological activity [14,15]. For a protein to exhibit E3 ligase activity, the RING domain must possess a proline residue after cysteine at the seventh position; this residue is missing in nematode LIN-41 and the protein lacks catalytic activity [16,17]. The crystal structure of the TRIM32 RING domain reveals a dimer of four alpha helices where both the N- and C-termini are located near the proximity of the core [18]. This dimerization promotes the association of E2-Ubiquitin conjugates and enhances the transfer of ubiquitin moieties. Another zinc-binding region is the B-box domain type II and, similar to the RING domain, this domain coordinates two zinc ions in a similar cross-brace manner. Typically, the B-box domain type II presents a two-turn α-helix followed by two short β-strands separated by a type-2 β-turn with two structured loops adjacent to the helix [19]. The B-box domains, together with the antiparallel coiled-coil region, allow for the formation of higher order complexes, but the domain itself is not crucial for catalytic activity. Instead, this region has been shown to influence the rate of ubiquitin chain assembly and contribute to the subcellular localization of TRIM32 [20].

Each NHL repeat is comprised of short stretches of about 40 amino acids. The X-ray structure of the TRIM32 NHL motif reveals a β propeller where each NHL repeat folds into four antiparallel β sheets arranged toroidally around a central axis (Figure 2B) [12,21]. These NHL repeats are essential for mediating protein–protein interactions and likely provide substrate specificity [2]. It has reported that the TRIM32 RING domain independently mediates dimerization and promotes tetramer formation [18]. This model predicts that oligomerization provides four substrate-recognition NHL domains that could enhance substrate binding and/or serve as a bridge to allow for substrates in close proximity. Additional biochemical approaches are required to validate the functional consequence of this structural arrangement.

### 1.2. E3 Ligase Activity and TRIM32 Substrates

**Figure 3:**
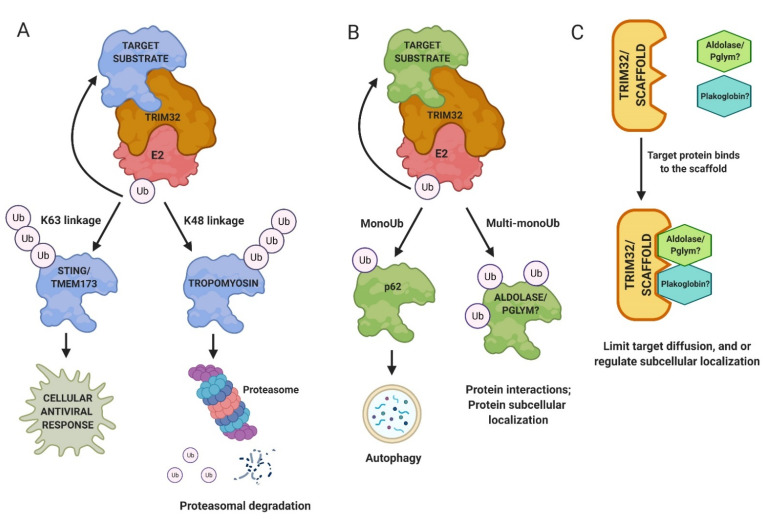
Mechanisms of TRIM32 function. (**A**) Polyubiquitination and proteasomal turnover of target substrates is a well-characterized role of TRIM32. (**B**) TRIM32 also appends single ubiquitin (Ub) molecules to proteins, resulting in either monoubiquitination or postulated multi-monoubiquitination. (**C**) Alternatively, TRIM32 may act as a scaffold protein to mediate protein localization within cells or to limit diffusion of proteins away for their sites of action.

Ubiquitination is a biochemical process that mediates numerous protein functions, including protein turnover, subcellular localization, and physical interactions within cells [22]. Ubiquitin is a small 8 kiloDalton (kDa) molecule that covalently attaches to lysine (K) residues on target proteins. Different types of ubiquitination on substrates lead to different fates [23]. Protein modifications can be either a single ubiquitin molecule (monoubiquitination) or a chain of ubiquitins (polyubiquitination). The first ubiquitin molecule covalently binds to a substrate through an isopeptide bond between the α-carboxyl group of the ubiquitin backbone and the ε-amino group of lysine on the target protein. Secondary ubiquitin molecules attach to one of the seven lysine residues of the previous ubiquitin molecule. This process requires the function of three classes of enzymes: an E1-ubiquitin activating enzyme (E1), an E2-ubiquitin conjugating enzyme (E2), and an E3 ligase. E3 ligases recruit the E2 enzyme, recognize the target substrate, and provide the catalytic domain that transfers the ubiquitin moieties to the substrate protein through a coordinated two-step enzyme reaction [24,25]. To clear out damaged, toxic, and/or misfolded proteins from tissues, cells undergo protein degradation, at least partly performed by 26S proteasome complex [26,27]. K48 polyubiquitin chains target proteins for the proteasome machinery, where the substrates are unfolded and proteolytically cleaved (Figure 3A) [28]. In other contexts, chains of K63-linked ubiquitin molecules can also trigger proteasomal degradation [29,30], but in the case of TRIM32, they mediate other functions, such as immune activation, thus far [31].

**Figure 4:**
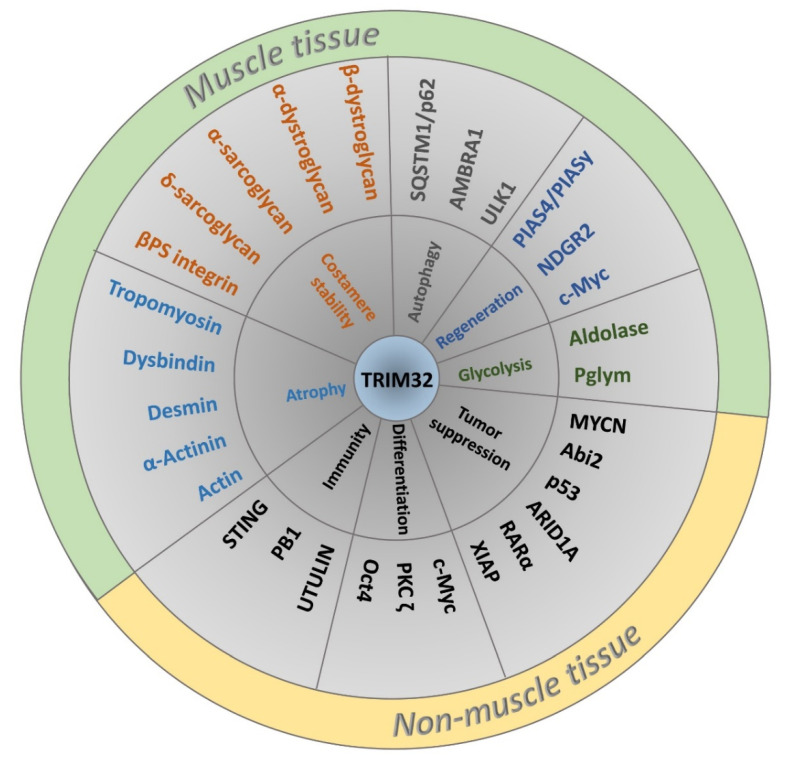
Classification of TRIM32 interacting proteins according to biological function and tissue specificity. The generalized role of TRIM32 in various biological processes is depicted in the inner circle followed by TRIM32 binding partners and/or substrates of E3 activity in the middle ring. Proteins with muscle-related functions are colored according to biological role.

The E3 ligase activity of TRIM32 is broad as it can ubiquitinate itself and many other substrates to regulate protein turnover and activity in different cell types (Figure 4; Supplementary Materials Table S1) [32]. Muscle substrates of TRIM32 include actin, tropomyosin, desmin, and dysbindin [33,34,35]. Abi2, c-Myc, MYCN, and p53 are all ubiquitinated by TRIM32 to regulate the cell cycle pathway in normal and/or cancerous cells [36,37,38,39]. Other substrates of TRIM32 include the anti-apoptotic component XIAP, an E3 SUMO ligase called Piasy, and the cytoplasmic enzyme NDRG2, among others [32,40,41]. Far less attention has been paid to multiple substrates that are monoubiquitinated by TRIM32, including NDGR2, TRIM72, actin, Piasy, and p62 [35,42,43]. One reason may be that the fate of monoubiquitinated proteins (e.g., protein trafficking or stabilization) is less defined (Figure 3B). It is also possible that TRIM32 functions as a scaffold, with roles that are independent of catalytic activity (Figure 3C).

## 2. Pathomechanism of TRIM32 in Limb-Girdle Muscular Dystrophy Type 2H

Limb-girdle muscular dystrophies (LGMDs) are a heterogeneous group of rare genetic disorders characterized by progressive muscle weakness and tissue degeneration [44,45]. Patients with LGMDs display mild-to-severe phenotypes with variable onset. Clinical features associated with LGMDs include difficulties in walking, climbing stairs, and scapular winging [2,46]. In some cases, disease progression is accompanied by respiratory and cardiac dysfunction. Genes that cause LGMDs can be inherited as dominant (LGMD1) or recessive (LGMD2) mutations. At present, 31 subtypes of LGMDs have been reported [2,46]. Currently, there are no treatments available to reverse muscle weakness or degeneration. However, supportive therapies can enhance quality of life.

### 2.1. Mutations Associated with TRIM32

Mutations in TRIM32 result in two clinically diverse diseases. The first is Bardet–Biedl syndrome (BBS), a genetically complex non-myopathic disorder involving retinal dystrophy, obesity, kidney abnormalities, and polydactyly [47]. A single mutation causative for BBS is in the B box region of TRIM32 [47]. LGMD2H, a mild autosomal recessive disorder, was first described in the Hutterite population of Manitoba, with TRIM32 identified as a causative gene associated with the dystrophy. The majority of LGMD2H-causing mutations cluster in the NHL repeats of TRIM32 (Figure 2C). The homozygous mutation p.D487N in the third NHL repeat was present in 41 Hutterite families with diagnosed LGMD2H [10,48,49]. Later, the same mutation was found in patients with sarcotubular myopathy (STM), indicating that these two disorders are subtle variants of one another [50]. Additional mutations in TRIM32 (p.R394H, p.T520TfsX13, and p.D588del) were found in European populations [33]. Patients with these mutations exhibit distinct clinical features, such as proximal muscle weakness, respiratory weakness, chronic keratitis, mild ankle contractions, and calf pseudohypertrophy [49,50].

Recently, four novel mutations in Spanish and Australian families have been detected in the RING, coiled-coil, or NHL domains of TRIM32 [51]. One family possessed a homozygous insertion, c.115_116insT (p.C39LfsX17), in the RING domain. In a separate family, the combined heterozygous mutations c.650A > G (p.N217S) and c.1701_1703del (p.F568del) emerged, involving the coiled-coil and fourth NHL repeat, respectively. The last characterized homozygous point mutation (p.V591M) is in the fourth NHL repeat of three patients from a single family. These patients with newly identified mutations showed variable clinical features such as ankle contractions, paravertebral muscle atrophy, and foot drop with proximal upper limb weakness. Magnetic resonance imaging (MRI) of muscle tissue revealed fatty degeneration in the thighs and lower leg and degeneration of the gluteus muscles, as well as the gastrocnemius muscles in the lower leg. Muscle biopsies from these patients exhibited reduced TRIM32 protein levels and increased autophagic flux. This suggests that the mutations have a crippling effect on TRIM32 protein. These variable phenotypes observed in LGMD2H patients highlight the importance of employing various models, each with their own advantages, to dissect the mechanisms behind disease pathology.

### 2.2. In Vitro Assessment of TRIM32 Function

One feature of nearly all TRIM proteins is the ability to self-associate. Indeed, an extended region of the TRIM32 RING domain forms a dimer in solution with or without the presence of the B-box domain [18]. Furthermore, this dimerization is essential for catalytic activity. Low resolution modeling based upon small-angle X-ray scattering (SAXS) experiments of the RING, B-box, and coiled-coil domains together support a model whereby the coiled-coil region can promote tetramerization of RING-induced TRIM32 dimers. Such an arrangement would provide four sites of the RING and NHL domains for substrate recognition or may allow for interactions with other binding partners. Other in vitro studies have shed light on how disease-causing mutations affect distinct biochemical characteristics of TRIM32. While the coiled-coil region is essential for forming higher-order complexes, the specific mutation in the B-box region causative for BBS (p.P130S) retains its dimerization ability [51] Other reports are conflicting, whereby some NHL domain mutations (p.R394H, p.D487N, and p.1559delC, but not D588del) have been shown to abolish the ability of TRIM32 to self-interact and homodimerize, while others have reported that the R394H and D487N mutants were still able to self-associate in yeast [33] Murine E2 enzyme UBE2N specifically binds TRIM32 and is crucial to catalyze the transfer of ubiquitin molecules to substrate proteins. The TRIM32 mutations p.D487N, p.R394H, and p.1559delC perturbed the ability of the protein to associate with its E2 enzyme [52]. Other experiments revealed that the D487N mutant was able to bind the human E2 ubiquitin-conjugating enzyme UbcM3 in HEK293T cells, but failed to monoubiquitinate TRIM32 [33]. These different results once again highlight the importance of implementing multiple systems to validate findings and/or to uncover cell or tissue differences.

### 2.3. Modeling of LGMD2H Point Mutations

LGMD2H pathological mutations cluster in the NHL domain and, intriguingly, the disease-causing amino acids R394H/R1114 and D487N/D1203 are conserved between human and fly TRIM32 protein [12,21]. Comparative thermostability analysis found that the R394H mutation has a lower melting temperature compared with the wild type (WT), indicating that this mutation renders the NHL domain unstable. Computational analysis of two point mutations, R394H and D487N, both show destabilizing ∆∆G. While there were no overall major structural changes observed in the NHL domain, there were subtle differences in certain regions of the protein where the WT and mutant proteins showed slightly distinct backbone conformations. The location of additional disease-causing point mutations retained a similar localization to R394H and D487N, where the mutations in either NHL1 (I395T) or NHL5 (V591M and R596G) are either just proximal or distal to a β strand, but largely internal within the entire NHL region (Figure 2B). Modeling of these additional point mutations largely supports a model whereby local and distant alterations within the NHL backbone structure promote NHL domain instability, thereby altering TRIM32 dimerization and/or interactions with other proteins, either E2 enzymes or target substrates.

TRIM32 shares significant structural and functional homology with malin, also an E3 ubiquitin ligase containing a RING domain and six NHL repeats [53]. Binding of the glucan phosphatase laforin to the NHL domains of malin targets this complex for the ubiquitination of substrates involved in glycogen metabolism [54,55]. Mutations in the genes encoding for malin (EPM2B) or laforin (EPM2A) result in Lafora disease, a form of progressive myoclonus epilepsy [56,57,58]. Mapping of the Lafora disease mutations in the NHL domain of malin shares a striking conservation with amino acids that are conserved in TRIM32 (21/37 amino acids) [59]. One of these conserved residues (D233 in malin) is equivalent to the D487 mutation in TRIM32, strongly arguing that particular amino acids are essential for either maintaining the overall structure of the NHL domain and/or preserving protein–protein interactions. Malin is not capable of ubiquitinating the TRIM32 substrates dysbindin or Piasy, but TRIM32 can ubiquitinate AMPK subunits, which are known malin target substrates. Interestingly, the topology of the appended ubiquitin chains differed, possibly due to the specificity of the E2 enzyme [60,61]. The malin ubiquitination complex added K63-linked polyubiquitin chains, while TRIM32 was capable of adding both K48- or K63-linked ubiquitin moieties. There are no reports of malin oligomerization, suggesting that the higher order structures required for TRIM32 catalytic activity may provide substrate specificity and contribute to the broad biological functions of TRIM32 compared with other E3 ligases.

### 2.4. TRIM32 Mouse Models for LGMD2H

TRIM32KO mice were the first in vivo model generated to study pathology associated with LGMD2H [62]. Disruption of TRIM32 resulted in animals with impaired muscle strength and reduced muscle weight. Ultrastructural and histological analysis in TRIM32-deficient muscles confirmed the presence of myopathic features such as centralized nuclei and fiber splitting. Transmission electron microscopy (TEM) analysis further unveiled extensive muscle damage, including Z-line streaming, autophagic vacuoles, myofibrillar degeneration, and a dilated sarcotubular system with prominent sarcoplasmic reticulum and mitochondrial defects [49,62]. These phenotypes recapitulated the clinical profile associated with LGMD2H and STM patients. mRNA profiling detected elevated expression of TRIM32 in the brain as compared with skeletal muscle, indicating a potential role for TRIM32 in the nervous system. Indeed, neurofilament proteins were decreased in TRIM32KO brains with reduced myelinated axon diameter. Along with muscle defects, the murine model also exhibits a neurogenic phenotype not observed in patients with muscular dystrophy [62]. To gain further insight into the myopathy mechanisms, a TRIM32 knock-in mouse was created that mimics the LGMD2H disease-causing D487N point mutation. Similar to TRIM32-null mice, TRIM32KI mice also presented mild myopathic and neurogenic phenotypes. The D487N point mutation reduced TRIM32 protein levels, suggesting that the mutation destabilizes the protein [63].

### 2.5. Drosophila Model for LGMD2H

The degenerative muscle mutant *lethal(2)thin (tn)* was identified as a recessive mutation in an ethyl methanesulfonate (EMS) screen performed in the fruit fly *Drosophila melanogaster*. Deficiency mapping narrowed down the candidate mutation to a small region on the second chromosome containing eleven candidate genes. Sequence analysis of *CG15105/another b-box affiliate (abba)* revealed itself as an orthologue of human TRIM32 [13]. In situ hybridizations showed robust *tn* mRNA expression in the developing embryonic musculature and protein localization at the Z-disc in larval muscles, in addition to the M-line in adult indirect flight muscles (IFMs) [11,13,21]. *tn* mutant embryos developed normally, but showed progressive muscle degeneration throughout larval development characterized by abnormal sarcomeric patterning and myofiber unbundling. These prominent defects were accompanied by functional locomotor deficits and *tn* homozygotes died during the pupal transition to adulthood. To gain an in vivo understanding of individual domain contributions in TRIM32 function, expression of TRIM32 variants that removed either the RING domain, B-box and coiled-coil regions, or NHL repeats were tested for their ability to rescue *tn* mutants. Deletion of any of these individual domains failed to prevent the severe striation defects and progressive muscle degeneration [11,21], emphasizing the requirement of each domain for normal TRIM32 function. These data are consistent with in vitro studies that the RING and coiled-coil domains are essential to form higher order complexes within cells [11,21]. Taken together, these abnormal muscle histology and mobility defects present upon loss of TRIM32 recapitulate phenotypes present in LGMD2H patients and provide a platform to further study disease pathogenesis. Moreover, the structural and functional conservation among proteins, including a majority of TRIM32 substrates, between fly and mammalian muscle tissue make the *Drosophila* LGMD2H model even more compelling for understanding molecular mechanisms that may contribute to disease initiation and/or progression.

The presence of both muscular and neuronal phenotypes in TRIM32 knockout (KO) mice suggested the possibility that the dystrophic muscles present upon loss of *Drosophila* TRIM32 could result from defective neuromuscular communication. However, abnormal locomotor ability and muscle degeneration were only present upon RNA interference (RNAi) of TRIM32 in muscle, but not in neuronal tissue [21]. Additional proof that the *Drosophila* model allows for the autonomous evaluation of TRIM32 muscle function came from experiments whereby expression of a full-length TRIM32 cDNA in muscle tissue rescued the degenerative *tn*-/- muscles [11,12,13]. This *Drosophila* larval muscle model also lacks adaptive immune cells and muscle stem cells, thus bypassing inflammation and complications associated with muscle tissue damage and repair.

**Figure 5:**
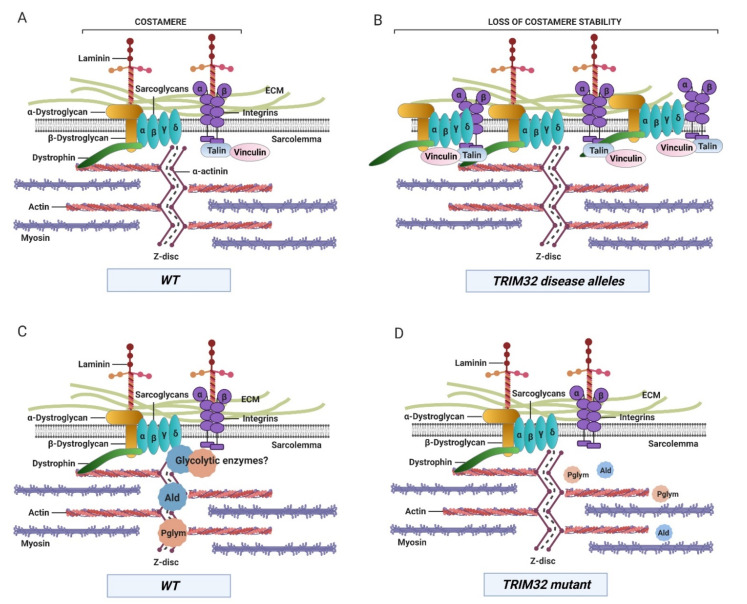
Compromising TRIM32 function results in the abnormal accumulation of costamere components and a decrease in glycolytic enzyme protein levels and/or localization within muscle tissue. (**A**) Large protein complexes called costameres connect the contractile apparatus at the Z-disc and sarcolemma to the extracellular matrix (ECM). (**B**) Both dystrophin glycoprotein complex (DGC) and integrin complexes are present at higher levels and lose their costamere location upon loss of TRIM32 or upon expression of LGMD2H alleles. (**C**) The glycolytic enzymes Ald and Pglym are found at the Z-disc in close association with costameres in normal muscle tissue. (**D**) Loss of TRIM32 reduces the levels of these proteins, either due to loss of their normal localization or lack of protein stability. WT, wild type.

Numerous studies have identified muscle proteins as targets of TRIM32 catalytic activity, including actin, tropomyosin, troponins, and α-actinin, during fasting conditions [34]. However, it is unclear if deregulation of any of these targets is sufficient to initiate or promote LGMD2H pathology. In *tn*-/- myofibers, actin, muscle LIM protein at 84B (Mlp84B), and α-actinin retained their normal Z-disc association [11,13]. In contrast, both the thick filament protein myosin heavy chain (MHC) and the thin filament protein tropomyosin (TM) were abnormally distributed along the sarcolemma. This evidence supports a role for TRIM32 in the structural maintenance of muscle, possibly in concert with sarcolemmal proteins. Candidates for such membrane proteins are Z-disc associated structures called costameres that physically link the sarcolemma to the internal muscle contractile apparatus and are essential for maintaining muscle integrity [64,65,66]. *Drosophila* possesses orthologs of the costameric proteins β-integrin, talin, spectrin, vinculin, and δ-sarcoglycan, suggesting conserved structure and function (Figure 5A). Microscopic analysis of these proteins in WT muscle showed localization along the sarcolemma at Z-disc junctions, confirming the existence of costameres in flies [11,13]. Loss of *tn* disturbed the spatial organization of examined costamere proteins, whereby these proteins were no longer retained at distinct costameres associated with the sarcolemma. These data strongly imply that TRIM32 is essential for costamere stability in *Drosophila*.

The generation of transgenic flies with targeted human mutations (R394H, D487N, and fs520) paved the way to uncover mechanisms associated with LGMD2H. Myopathic expression of each of these pathological alleles exhibited larvae with muscle defects and reduced TRIM32 protein levels similar to patients with LGMD2H [21]. Further examination of specific muscle proteins revealed a surprising discovery. Assessed by Western blotting, the total protein levels of TM, β PS integrin, and sarcoglycan δ were elevated upon loss of TRIM32. However, expression of the LGMD2H disease-causing alleles did not alter levels of TM, but only the costamere proteins β PS integrin and Sarcoglycan δ (Figure 5B). These data highlight two important features of TRIM32 function in muscle and possibly disease progression. First, distinct subcellular populations of TRIM32 may influence target substrate specificity because only costamere proteins are upregulated upon expression of human disease alleles. Second, the increased levels of α-dystroglycan, β-dystroglycan, and α-sarcoglycan upon expression of a dominant-negative version of TRIM32 in murine C2C12 cells also suggest that this TRIM32-mediated regulation of mammalian costamere is conserved across species [21]. Further extending the connection between TRIM32 and costamere stability, a recent report structurally and functionally links the dystrophin glycoprotein complex (DGC) to the insulin receptor in mouse skeletal muscle [67]. Because TRIM32 reduces insulin pathway activity [68], this may also contribute to dampened DGC/costamere stability.

## 3. The Complicated Role of TRIM32 in Muscle Homeostasis

Skeletal muscle fibers may undergo physiological changes as a consequence of metabolism, age, preservation of homeostasis, and/or in response to altered myogenic signals, often resulting in pathological conditions. A prominent feature of dystrophic muscle is atrophy, or progressive muscle wasting, whereby the delicate balance between protein synthesis and degradation is shifted towards overall reduced muscle protein content [69,70,71]. Two major pathways that regulate protein turnover, and thus control muscle mass, are the ubiquitin-proteasome system (UPS) and the autophagy-lysosome system (ALS). Balancing this degradation of muscle proteins is the regeneration of muscle tissue by satellite cells. Both atrophy and regeneration are discussed below in the context of TRIM32, but are reviewed in detail in Lazzari and Meroni [7].

### 3.1. Atrophy

Insulin (or IGF-1)→Akt is one of the primary signaling pathways responsible for muscle growth [72,73]. Activation of phosphatidylinositol-3-kinase (PI3K) generates the membrane phospholipid phosphoinositide-3,4,5-triphosphate (PIP3). PIP3 acts as a docking site for phosphoinositide-dependent kinase 1 (PDK1) and Akt. PDK1 phosphorylates Akt and leads to its activation. Further activated Akt represses the transcription factor FOXO and stimulates protein synthesis essential for muscle growth. Conversely, FOXO activation during fasting induces the expression of atrophy-related genes and ubiquitin ligases, which control the rapid degradation of myofibrillar components [74].

The Goldberg lab recognized the role of TRIM32 in regulating insulin→PI3K→Akt→FoxO signaling in both normal and atrophying muscles [68,75]. Plakoglobin, a component of the desmosome adhesion complex, regulates cell motility, growth, and differentiation [76,77]. During fasting, TRIM32 reduced the association of plakoglobin with the p85 subunit of PI3K, which negatively influenced PI3K/Akt signaling. However, plakoglobin levels were not affected in fasting/atrophying muscles, proposing a mechanism of regulation different than proteasomal degradation (Figure 3C). Downregulation of plakoglobin levels in normal muscles led to decreased phosphorylation of PI3K, Akt, FOXO3, and the subsequent atrophy of muscles. This PI3K/Akt signaling involving TRIM32 and plakoglobin provided a new mechanism of muscle growth [67,68].

Acute muscle atrophy induces rapid degradation of sarcomeric proteins by several E3 ubiquitin ligases such as muscle ring finger 1 (MURF1) and muscle atrophy F box (MAFbx, also called Atrogin1) [78]. shRNA-mediated knockdown of TRIM32 in mouse muscle during fasting attenuated muscle wasting and loss of actin, tropomyosin, and troponin; however, TRIM32 expression was not induced unlike other atrophy-related E3s, MAFbx/atrogin-1, and MuRF1/TRIM63 [34,78]. In an effort to identify new substrates of TRIM32, muscle extracts incubated with TRIM32 precipitated thin filament components (actin and tropomyosin) and Z-disc associated proteins (desmin and α-actinin). In vitro ubiquitination assays performed on muscle extracts provided evidence that TRIM32 ubiquitinated actin [35] and tropomyosin [34], and regulated the Z-disc associated protein desmin, an intermediate filament protein crucial for the integrity of thin filaments. During fasting, desmin filaments underwent rapid phosphorylation, which promoted ubiquitination by TRIM32 and the disassembly of thin filaments. Interestingly, cell- and/or tissue-specific regulation of TRIM32 activity may account for differences observed in fasting experiments. Whole body KO of mouse TRIM32 did not reveal substantial differences in body weight, muscle mass, or the accumulation of ubiquitinated proteins upon fasting-induced atrophy [41]. It is possible that complete deficiency of TRIM32 during development elicits compensatory responses by additional E3 ligases, whereas the selective transient downregulation of TRIM32 in normally developed adult mouse muscle does play a role in atrophy.

Other data demonstrate that TRIM32 induces autophagy as a protective mechanism to attenuate muscle damage during atrophy. The activating molecule in BECN1-regulated autophagy protein 1 (AMBRA1), a positive regulator of autophagy, was identified in a proteomic screen with TRIM32. Further domain analysis confirmed that AMBRA1 binds to the catalytic RING domain of TRIM32. Downregulation of AMBRA1 levels in C2.7 myoblasts reduced basal autophagic flux, while knockdown of TRIM32 had no effect [79]. TRIM32-deficient myotubes treated with dexamethasone, a synthetic analog of glucocorticoids, both induced atrophy and impaired autophagy, assayed by reduced protein levels of LC3-II, indicative of defects in autophagic flux. Thus, TRIM32 impaired autophagy in atrophic cells. Further experiments showed that TRIM32 binds Unc-51 like autophagy activating kinase 1 (ULK1), an autophagy regulator in complex with AMBRA1 through the RING domain. In vitro ubiquitination assays proved that TRIM32 modifies ULKI and adds K63 linked polyubiquitin chains essential for the kinase activity of ULK1. Dexamethasone treatment of HEK293T cells expressing the TRIM32 pathogenic mutants D487N and R394H mutants were unable to physically interact with ULK1 to promote autophagy. Final confirmation of a role for TRIM32 in autophagy was obtained in LGMD2H patients, who also showed defects in autophagy upon atrophic stimulation [79].

### 3.2. Regeneration and Growth

Adult skeletal muscle has a remarkable regenerative capacity in response to injury, aging, and/or inflammation. Skeletal satellite cells are quiescent myogenic cells located between the sarcolemma and basal lamina, and can become activated to facilitate muscle repair [80,81]. Early studies established that TRIM32 expression increased about 2-fold during muscle unloading and 4.5-fold during muscle reloading in a hindlimb suspension model [35]. This increased protein expression argues for a role for TRIM32 in muscle regrowth after atrophy or disease. Indeed, TRIM32 is selectively upregulated in regenerating fibers derived from Duchenne muscular dystrophy (DMD) or Becker muscular dystrophy (BMD) patients, further highlighting the importance of TRIM32 in muscle growth [82].

TRIM32 is expressed in adult skeletal muscles, including regenerative myofibers, but also in primary myoblasts and in satellite cells [63,83]. Functionally, TRIM32 KO myoblasts show impaired differentiation and premature senescence, marked by elevated levels of the cell senescence markers and the E3 SUMO ligase PIAS4 [41]. Based upon the rationale that additional substrates of TRIM32 would be increased upon loss of E3 activity, 2D fluorescence difference gel electrophoresis (2D-DIGE) followed by MS identified N-myc downstream-regulated gene (NDGR2) as an additional TRIM32 substrate [42]. Polyubiquitination of NDGR2 was decreased in TRIM32KO myoblasts, resulting in an accumulation of NDGR2. Of note, overexpression of NDGR2 in myoblasts slowed the rate of cell proliferation and delayed cell cycle withdraw. Further supporting a role for TRIM32 in differentiation, the ubiquitination and degradation of c-Myc that is mediated by TRIM32 regulates myoblast differentiation and muscle regeneration [83]. Taken together, these findings provide a strong basis for the requirement of TRIM32 in muscle growth, which will be expanded upon below.

## 4. TRIM32, Glucose Metabolism, and Link to Cancer

Glycolysis is a universal central metabolic pathway involved in energy production required for cellular activities. Although glycolysis is less efficient than oxidative phosphorylation in yielding net ATP, metabolic intermediates of the glycolytic pathway are essential for synthesizing macromolecules necessary for cell survival, signal transduction, and biomolecular interactions [84]. Reprogramming of glycolysis controls carbohydrate metabolism in both skeletal muscle and various cancer types [85,86]. The ubiquitous expression of TRIM32 makes it a reasonable candidate to be implicated in muscle function as well as tumor suppression and/or progression. This section summarizes the role of glycolysis and TRIM32 in muscle development and tumorigenesis and establishes a functional relationship between TRIM32 and metabolism.

### 4.1. Glycolytic Enzymes in Muscle Physiology

Aside from movement, additional functions of skeletal muscle ensure oxygen consumption, nutrient storage, and regulation of whole body metabolism. Therefore, maintaining skeletal muscle strength and structure is vital to maintain quality of life and to ensure proper musculoskeletal function. Muscle mass adapts to pathophysiological changes in the body, and defects in anabolic signaling are prominent in aging, disuse, malignancies, and injury, which promote loss of muscle mass and strength [87]. Various genetic models (mice, flies, and worms) have been employed to study the mechanisms associated with skeletal muscle metabolism and physiology.

Skeletal muscle comprises ~40–50% of the total body weight and mainly derives energy from glucose and fatty acids [88]. To ensure efficient muscle performance, a constant supply of ATP is crucial. Vertebrate muscle fibers are categorized into three main types based upon their ability to produce ATP. Fast glycolytic fibers primarily feed on anaerobic glycolysis as their source of ATP and have fast contractions. These fibers store a large amount of glycogen used in glycolysis to generate ATP quickly. Other types of fibers are slow oxidative (SO) and fast oxidative (FO), where SO fibers primarily have slower contractile speeds and utilize aerobic respiration with fatty acids as a primary source to meet the demands of contracting muscle. In contrast, FO fibers use aerobic respiration, but can switch to glycolysis for energy and can fatigue more quickly than SO fibers. However, glycolytic fibers produce less ATP than oxidative fibers [89,90]. As individual fiber types within the same whole muscle may show differential responses, it is important to understand how metabolism, protein synthesis, and cell growth are coordinated.

**Figure 6:**
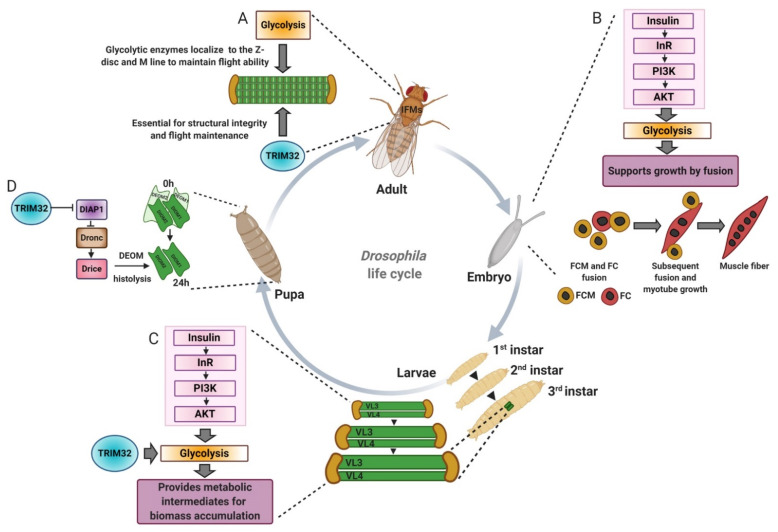
The *Drosophila* model has been instrumental in understanding how TRIM32 and glycolysis regulate cell growth and cell death. (**A**) Glycolytic enzyme localization to the Z-disc and M-line in *Drosophila* adult indirect flight muscles (IFMs) is required for flight maintenance. TRIM32 is also required to maintain the structural integrity of the IFMs, possibly by mediating the subcellular distribution and/or stability of glycolytic enzymes. (**B**) Insulin acts upstream of glycolysis to promote muscle growth by fusion during embryonic development. (**C**) TRIM32 directly acts on glycolytic enzymes to regulate protein levels and/or protein localization, which in turn allows for high glycolytic flux in larval tissues undergoing massive cell growth. (**D**) Much like that of XIAP, TRIM32 regulates the levels of DIAP1 to control the apoptotic death of a set of muscles that undergo histolysis during pupal development. DEOM, dorsal external oblique muscle. FCM, fusion competent myoblasts. FC, founder cells.

Components of the glycolytic pathway provide ATP critical for the contractile apparatus within muscle fibers. Antibody staining in the primarily oxidative adult *Drosophila* indirect flight muscles reveals a precise, defined localization for the glyceraldehyde-3-phosphate dehydrogenase (GAPDH), aldolase (Ald), triosephosphate isomerase (TPI), and phosphoglycerate kinase (PGK) at the M-line and Z-disc within the sarcomere (Figure 6A) [91,92]. *Drosophila* Gpdh mutants are defective in flight, signifying the requirement of glycolytic enzymes for function. Further, myofibrils from Gpdh null mutants altered the localization of other glycolytic enzymes (GAPDH, aldolase, TPI, and PGK) at the M-line and Z-disc, providing evidence that these enzymes are interdependent and form complexes at the sarcomere [92]. Functional advantages of glycolytic subcomplexes include limiting substrate diffusion and efficiently channeling substrates for energy production [93]. In mammalian striated muscle, nearly all glycolytic enzymes are located at the I-band and possibly the Z-disc [93,94,95]. The mechanisms behind the colocalization of glycolytic enzymes within the sarcomere are not clear, but numerous studies suggest that glycolytic enzymes interact with F-actin or troponin. Strong evolutionarily conservation is evident, even in Saccharomyces cerevisiae, which lacks muscle sarcomeres, where physical interactions between glycolytic enzymes and F-actin are observed [96,97]. Additional experiments are required to understand how these enzymes localize at the M-line and Z-disc and provide ATP for muscle contraction.

### 4.2. Drosophila as a Model for Glycolytic Growth

Studies performed in *Drosophila* highlight the importance of glycolytic proteins in normal muscle development. Jagla and colleagues screened for orthologous genes in *Drosophila* and zebrafish to study their function in muscle homeostasis [98]. Gene ontology analysis revealed enrichment of genes encoding metabolic proteins, including those involved in the glycolytic pathway. Phosphoglycerate mutase 78 (Pglym78) mutants exhibited an altered embryonic muscle architecture, whereby developing muscles were reduced in size with fewer nuclei indicative of myoblast fusion defects. Actin foci formation, a prominent feature of fusion-competent myoblasts, was also affected in Pglym78 mutants. Similarly, attenuating the expression of vertebrate orthologue Pgam2 in zebrafish showed reduced birefringence and perturbed muscle architecture. Muscle-specific reduction of the additional glycolytic genes Pfk, Tpi, Gapdh1, Pgk, pyruvate kinase (PyK), and lactate dehydrogenase (Ldh/Impl3) presented embryos with thinner muscles. Expression of a dominant negative form of the insulin receptor (InRDN) or reducing muscle specific expression of Akt showed embryos with thinner muscle and defects in myoblast fusion similar to the phenotype observed in the glycolytic gene deficient embryos pathway (Figure 6B). Together, these in vivo genetic data affirm the idea of the involvement of glycolytic genes in promoting myogenic function in coordination with growth due to induction of the insulin/Akt pathway [98,99].

Apart from serving as a source of energy, glycolysis provides building blocks to support the massive growth of tissues, especially muscle, during *Drosophila* larval development. This massive increase in muscle fiber size occurs without the addition of new myoblasts and is partially regulated by the insulin/Tor pathway followed by nuclear growth via endoreplication [100,101]. Another major contributing factor to cell growth involves glycolysis, whereby glycolytic intermediates serve as precursors for the synthesis of amino acids, fatty acids, and nucleotides to support biomass production [84]. The Tennessen lab identified the estrogen-related receptor (dERR) as a key regulator of carbohydrate metabolism throughout muscle development. Microarray analysis performed on dERR mutants indicated downregulation of glycolytic genes and metabolomic studies confirmed a functional deficit in carbohydrate metabolism, including a reduction in lactate levels [102]. Elevated levels of lactate hydrogenase (LDH) convert pyruvate to lactate and permit the regeneration of NAD+ levels required to maintain high rates of biomass production [103]. This upregulation of LDH in larval muscles, combined with the dERR-mediated regulation of carbohydrate metabolism genes, ensures sufficient glycolytic intermediate precursors to support the dramatic growth of larvae post embryogenesis [12,104,105]. With increased muscle growth, new sarcomeres are added; however, molecular mechanisms coordinating new sarcomere addition and cell growth are just beginning to be understood [106].

Altered metabolism is often associated with myopathies and muscular dystrophies [107]. Integration of metabolomics and proteomics approaches has uncovered dramatic changes in diseased muscle tissue. Proteomic profiling of skeletal muscle proteins showed elevated levels of PGK, ENO, ALD, TPI, and LDH in aged mice [108]. In Duchenne muscular dystrophy (DMD), glycolytic type II fibers are preferentially affected over oxidative type I fibers with significant cardiac dysfunction, which suggests cardiac atrophy and remodeling [109,110]. Patients with DMD exhibit dysregulated glycolytic metabolism with a reduction in phosphoglycerate mutase (PYGM), aldolase A (ALDOA), cytoplasmic glycerol-3-phosphate dehydrogenase (GPD1), triosephosphate isomerase (TPI1), phosphoglycerate kinase (PGK1), beta enolase (ENO3), and pyruvate kinase M1/M2 (PKM2) enzymes [111]. Similarly, glycolytic fibers are more prone to atrophy induced by cancer, sepsis, fasting, and glucocorticoids [109] and proteomic characterization performed on muscle biopsy specimens of patients with inclusion body myositis also showed perturbed abundance of glycolytic enzymes [112,113]. These studies suggest that, in different pathological conditions, while altered metabolism may be a secondary consequence, a better understanding of metabolic changes that initiate and/or promote disease progression may lead to novel treatment options.

The inherent glycolytic nature of *Drosophila* larval muscles shares the same metabolic profile as type II fibers affected in muscle diseases and provides a unique model to study muscle metabolism and disease mechanisms [102,114]. Previous studies in the Geisbrecht and Cripps labs found that loss of *Drosophila* TRIM32 exhibited smaller muscles with progressive tissue degeneration [13]. It was assumed that this reduced cell size was a secondary consequence of muscle deterioration. However, the surprising discovery was made that TRIM32 physically interacts with two enzymes that function in glycolysis [12]. An in vivo proteomics approach to identify proteins that physically interact with the NHL domain of TRIM32 detected an enrichment in glycolytic enzymes. In vitro binding assays and co-immunoprecipitation analysis confirmed binding of two of these enzymes, Ald and Pglym78, with TRIM32 (Figure 5C). Notably, the sarcomeric localization of both Ald and Pglym78 was also lost in *tn*-/- muscle tissue (Figure 5D).

While TRIM32 can mono- and poly-ubiquitinate proteins, the majority of studies have focused on the biological impact of K48 polyUb chains in the proteasomal turnover of substrates. Strikingly, rather than the expected increase in protein levels upon loss of an E3 enzyme, loss of TRIM32 caused a ~50% decrease in Ald or Pglym protein levels. Metabolomic analysis of *tn* mutant larvae also confirmed a decrease in glycolytic flux, substantiated by lower levels of the terminal glycolytic products pyruvate and lactate, in addition to a decrease in the glucose-derived metabolites glycerol-3-phosphate and 2-hydroxyglutarate. Confirmation that the reduced muscle size present upon loss of TRIM32 was a consequence of perturbed muscle glycolysis was solidified by findings that muscle-specific expression of dERR in *tn*-/- muscles was sufficient to ameliorate loss of muscle and to stabilize Ald and Pglym78 protein levels. These data confirm that TRIM32 is required for glycolytic protein stability and/or subcellular localization in muscle tissue (Figure 3B,C). The downregulation of several glycolytic proteins, including GAPDH and PyK, derived from murine Trim32KO muscle tissue [42], strongly suggests conserved mechanisms mediating homeostasis in both *Drosophila* and mammalian muscle.

Mechanistic insight into treatment options for the restoration of muscle mass emerged after further metabolomics analysis [12]. There was a statistically significant reduction in the overall levels of 11 of the 20 amino acids upon loss of TRIM32. Dietary supplementation of *tn* mutants with either yeast extract containing all macromolecules or simply the 20 individual amino acids improved muscle mass. Altogether, these data argue that *tn* mutant muscles are uniquely sensitive to dietary amino acids, and further confirm that glucose metabolism is used to synthesize amino acids and other metabolites for cell growth (Figure 6C).

### 4.3. TRIM32 Function as a Tumor Suppressor

Tumor cells evade apoptosis and continue to proliferate uncontrollably. Programmed cell death is a major targeted pathway for tumor suppression, and various chemotherapeutic agents induce apoptosis to slow the spread of cancerous tissues [115,116]. TRIM32 is an intriguing protein that regulates both cell death and survival pathways. X-linked inhibitor of apoptosis protein (XIAP), one of the components of anti-apoptotic machinery, is often overexpressed in malignant tumors and is an attractive target for cancer therapy [117,118,119,120,121,122]. TRIM32 is an E3 ubiquitin ligase for XIAP and downregulates the anti-apoptotic pathway [40]. Overexpression of TRIM32 in HEK 293T cells reduces XIAP protein levels and facilitates *TN*Fα-sensitized cell death through the ubiquitination of XIAP. *Drosophila* TRIM32 genetically interacts with a number of genes that regulate apoptosis, including death-associated inhibitor of apoptosis (DIAP1), the vertebrate orthologue of XIAP, death regulator Nedd2-like caspase (Dronc), and death-associated APAF1-related killer (Dark). Loss of TRIM32 increased DIAP protein levels, suggesting a mechanism similar to that of XIAP, in ubiquitination-mediated degradation. Functionally, elevated DIAP levels blocked processing of the initiator caspase Dronc, and prevented degradation of the larval dorsal external oblique muscle (DEOM) abdominal muscles (Figure 6D) [123]. These results not only mimic the apoptotic regulation via vertebrate TRIM32 to confirm functional conservation between two species, but also extend commonalities of TRIM32 function in normal muscle tissue and cancer cells.

Another tumor suppressor function of TRIM32 was defined in its ability to facilitate the proteasomal degradation of MYCN in neuroblastoma cells [38]. The inability to maintain asymmetric cell division in *Drosophila* neuroblasts or human neuroblastoma cells may lead to tumorigenesis [124]. TRIM32 recruitment to the spindle poles promoted MYCN degradation, which is essential for the establishment of asymmetric cell divisions [38]. Amplification of the MYCN oncogene has been observed in retinoblastoma, glioblastoma, and medulloblastoma tumors [125], and raises the possibility that TRIM32 plays a broader role in tumor suppression than previously thought. It is worth noting that TRIM32 also ubiquitinates and degrades c-myc, necessary for muscle stem cell differentiation [83]. Taken together, the data suggest that TRIM32-mediated targeting of myc proteins may provide new avenues for stem cell cancer drug therapy.

### 4.4. TRIM32 and Its Oncogenic Role in Tumor Growth

TRIM32 mRNA and/or protein is upregulated in benign and malignant tumors, suggesting a potential role for this E3 ligase in tumor progression [36,126,127,128,129]. In response to stress, p53 induces the transcriptional expression of TRIM32 [37]. Subsequent binding of TRIM32 to p53 through its NHL domain negatively regulates p53 protein turnover through the proteasomal degradation pathway, effectively blocking p53-mediated apoptosis and promoting xenograft tumors. Elevated TRIM32 mRNA levels are also observed in epidermal carcinogenesis models. Further, studies confirmed that TRIM32 induces epidermal thickening in vivo and negatively regulates apoptosis in *TN*Fα/UVB mediated oncogenic response in keratinocytes, and promotes cell survival through degradation of PIASY [126]. Under normal cellular conditions, Ab1 interactor 2 (Abi2) suppresses cell growth and reduces cell motility. TRIM32 also ubiquitinates and degrades Abi2, and promotes cell growth and proliferation [36]. Qingyu Luo and colleagues found that TRIM32 mRNA levels are elevated in head and neck squamous carcinomas [130]. Squamous cell carcinomas (SCCs) are aggressive, and patients with SCCs have low ARID1A (AT-rich interaction domain 1A) expression, resulting in poor prognosis. Screening of E3 ligases in 293T cells identified TRIM32 as a regulator of ARID1A protein levels, whereby excess TRIM32 promotes SCC proliferation and chemoresistance by degrading ARID1A [130]. TRIM32 overexpression promotes lung cancer cell proliferation by activating the JAK2/STAT3-signaling pathway [131], as STAT3 has also been implied in muscle wasting during cancer [132], it would be interesting to test if TRIM32 also activates this inflammatory pathway in muscles. Elevated expression of TRIM32 in non-small cell lung cancer (NSCLC) cell lines induced cisplatin chemoresistance. Cell-based assays showed TRIM32 upregulates mitochondrial membrane potential, increases cell proliferation, and reduces cisplatin-induced ROS accumulation. Further, an increase in expression of Bcl2, a known suppressor of mitochondrial apoptosis, was observed upon TRIM32 induction [133].

There is decades of evidence that some tumor cells favor glucose metabolism to generate energy and metabolic intermediates for the production of cellular constituents to support cell proliferation and growth. This metabolic reprogramming, termed the Warburg effect, is characterized by high rates of glycolysis concomitant with increased glucose uptake and lactate production [84,86]. The elevated glycolytic rate that operates in *Drosophila* larval muscle is analogous to the Warburg effect in rapidly proliferating cancer or stem cells, where glucose metabolism is diverted towards the generation of amino acids [134]. As mentioned previously, the Geisbrecht lab uncovered that loss of TRIM32 reduces glycolytic flux, thus limiting the ability of cells to produce cellular building blocks required for muscle growth [12]. Loss of TRIM32 also negatively impacted glycolysis in two other tissues with increased LDH activity and elevated glycolytic rates, the larval brain and wing disc-derived epithelial tumors. Knockdown of TRIM32 specifically in neuronal tissue reduced the overall size of the larval brain and compromised glycolytic flux, substantiated by a decrease in L-lactate and proton efflux. Additional experiments revealed that this deficiency of TRIM32 in brain tissue did not alter cell proliferation or apoptosis, but reduced overall cell size, consistent with a positive role for TRIM32 in cell growth via biomass accumulation. Activation of *Drosophila* PDGF/VEGF-receptor (Pvr) in the imaginal discs causes epithelial tumors with induced aerobic glycolysis and upregulated LDH activity [135]. Growth of these tumors in *tn* mutants that lack TRIM32 was dramatically reduced [12]. These results confirm a novel role for TRIM32 for glycolytic-mediated cell growth in both normal and tumor tissues and may pave the avenue for further studies in TRIM32KO mice to also understand if they may develop spontaneously less tumors with aging than their wild-type counterparts.

The PI3K/Akt pathway enhances glycolysis in various cancer cells and promotes apoptosis and cell proliferation in gastric cancer cells [136,137,138,139,140]. TRIM32 upregulation poorly correlates with the overall survival of patients with gastric cancer. Jianjun Wang and colleagues identified a potential link between TRIM32 and Akt signaling in gastric cancer cells. Knockdown of TRIM32 inhibited phosphorylation of Akt in NCI-N87 and MKN74 cells and suppressed the growth of the cells. GLUT1 and HKII protein levels were also reduced upon TRIM32 silencing. In contrast, overexpression of TRIM32 improved the phosphorylation of Akt and inhibited apoptosis. TRIM32 is expressed in all cells of the human body and, therefore, may be a general regulator of growth in normal and cancerous tissues that require a rapid increase in cell size [141]. A better understanding of TRIM32 function may lead to therapeutic interventions to either increase cell size in patients with mutations in TRIM32 or to limit the growth of cancers that overexpress TRIM32.

Multiple other reports also link other TRIM family proteins to tumor growth via glycolysis. Sub-class C-VI family member TRIM24 is significantly upregulated in breast cancer and promotes a signature characteristic of glycolytic gene expression [142]. Glucose uptake is enhanced in these cells with a corresponding increase in basal glycolysis and basal mitochondrial respiration, although a molecular mechanism is not yet known. In a different scenario, mechanical regulation of the actin cytoskeleton regulates glycolysis via TRIM21 (sub-group C-IV)-mediated ubiquitination of phosphofructokinase (PFK) [143]. Bundling of F-actin filaments or stress fiber formation forces the sequestering of TRIM21 to allow for high glycolytic flux through the upregulation of glycolytic enzymes and subsequent growth of transformed NSCLCs. Involvement of the cytoskeleton in controlling glycolytic activity and TRIM32 function is an interesting link because there is ample evidence that glycolytic proteins bind to actin and possibly TRIM32 in muscle tissue [12,93].

While other ubiquitin-ligases function as one component of a multisubunit Skp1-Cullin1-F-box protein (SCF) ubiquitin ligase complex (e.g., MAFbx/atrogin-1) [144], TRIM32 and other TRIM proteins function as simple RING E3 ligases, which can exist as monomers, dimers, or tetramers [2,5]. This monomeric structure makes it more “druggable” to design possible TRIM32 inhibitors, as some have already been identified for other members of this family (e.g. TRIM63/MuRF1 inhibitors are currently under testing against muscle atrophy [145,146], among others better described [147]. It is possible that TRIM32 inhibitors could work as anticancer agents.

## 5. Conclusions and Future Directions

It has become increasingly clear that multiple model systems, each with its own advantages, are essential to understand the pleiotropic roles of TRIM32 in development, homeostasis, and disease. The ubiquitous expression of TRIM32 in mammalian systems has made it challenging to identify target substrates, but also to dissect how mutations in different regions of TRIM32 cause different pathological diseases. Use of the *Drosophila* larval model allows for the study of TRIM32 function in muscle homeostasis, mainly in the prevention of tissue degeneration, without complications of a neuronal contribution or immune cell infiltration, the latter of which may drive disease progression. Furthermore, the ability to cause progressive muscle deterioration in larval or adult muscle upon expression of human pathogenic alleles not only established a muscle-autonomous role for these mutations in LGMD2H progression, but also confirmed the importance of costamere protein stability in disease prevention. In contrast, the lack of satellite cells in *Drosophila* larval muscle tissue prevents a complete understanding of TRIM32 function in tissue repair and regeneration. It would be interesting to test if LGMD2H mutations promote disease progression in mice models or if the role of TRIM32 in satellite cells is independent of the NHL domain.

Multiple experimental results highlight aspects of TRIM32 structure and function that are conserved not only between the *Drosophila* and mammalian protein, but also across different cell types. *Drosophila* TRIM32 promotes cell growth in normal and tumor tissues that exhibit elevated glycolytic flux [12]. Therefore, changing the metabolic balance within cells towards anabolic pathways may be an underlying mechanism for abnormal cell growth in multiple tumor types that overexpress TRIM32. Common target substrates are also shared across species. TRIM32 mediates DIAP1 levels to prevent muscle cell apoptosis during pupal development [123], while XIAP is similarly targeted by mammalian TRIM32 to prevent apoptosis of cancer cells [40]. The role of TRIM32 in enhancing glycolytic flux is aligned with its role in promoting cancer, but conceptually contradicts Trim32’s role in inhibiting cancer growth. One possibility to explain this discrepancy is rapid rewiring of metabolism in response to anabolic or catabolic signals to promote or reduce cell growth, respectively. An important aspect of this rewiring model involves the access of TRIM32 to different protein substrates, illustrated by the numerous targets of TRIM32 identified to date (Figure 4; Table S1). A better understanding of TRIM32 activity, not limited to polyubiquitination, but including other types of ubiquitin modifications, as well as scaffolding functions, will provide more insight into how an ubiquitous protein can regulate proteins in different subcellular compartments of all cells to produce distinct biological outputs.

## Data Availability

Data are contained within the article.

## Supplementary Materials

The following supplementary data are available online at https://www.mdpi.com/2218-273X/11/3/408/s1. Table S1: TRIM32 substrates.
