# Supplementary material for: TRIM32: A Multifunctional Protein Involved in Muscle Homeostasis, Glucose Metabolism, and Tumorigenesis
Source: Biomolecules. 2021 Mar 10;11(3):408. doi: 10.3390/biom11030408 (PMC7999776; doi:10.3390/biom11030408)
Supplement: Supplementary file 1 [file biomolecules-11-00408-s001.pdf]

Table S1. TRIM32 substrates

TRIM32 substrates (gene)	Model	TRIM32 mutation(s)	Biological process affected (Tissue)	Mono- or polyUb (verified linkage)	Reference
α-Actin (Acta)	In vitro; mouse TA muscle[1]	shTRIM32	Atrophy (fasting muscle)	Mono- & polyUb	Kudryashova, et al., 2005 Cohen, et al., 2012
α-Actinin (Actn3)	In vitro; mouse TA muscle	shTRIM32	Atrophy (fasting muscle)	Not tested	Cohen, et al., 2012
Desmin (Des)	In vitro; mouse TA muscle	shTRIM32	Atrophy (fasting muscle)	PolyUb	Cohen, et al., 2012
Tropomyosin1 α chain (Tpm1)	In vitro; mouse TA muscle	shTRIM32	Atrophy (fasting muscle)	Mono- & polyUb	Cohen, et al., 2012
Dysbindin (DTNBP1)	HEK293T cells	R394H; D487N	Atrophy (muscle denervation)	PolyUb	Locke et al., 2009
Tropomyosin 2 (Tm2)	*Drosophila*	TRIM32 mutant	Muscle	Not tested	LaBeau-Dimenna, et al., 2012; Bawa, et al., 2020
βPS integrin (mys)	*Drosophila*	R394H; D487N; fs250	Costamere stability (muscle)	Not tested	LaBeau-Dimenna, et al., 2012; Bawa, et al., 2021
sarcoglycan δ (Scg δ)	*Drosophila*	R394H; D487N; fs250	Costamere stability (muscle)	Not tested	LaBeau-Dimenna, et al., 2012; Bawa, et al., 2021
α-sarcoglycan (SGCA)	C2C12 cells	TRIM32ΔRING	Costamere stability (muscle)	Not tested	Bawa, et al., 2021
α-dystroglycan (DAG1)	C2C12 cells	TRIM32ΔRING	Costamere stability (muscle)	Not tested	Bawa, et al., 2021
β-dystroglycan (DAG1)	C2C12 cells	TRIM32ΔRING	Costamere stability (muscle)	Not tested	Bawa, et al., 2021
Sequestosome-1/p62 (SQSTM1)	HEK293 cells	TRIM32_C44S; TRIM32D487N	Autophagy (atrophic muscle)	MonoUb (K48 & K63)	Stange-Overå, et al., 2019
BECN1-regulated autophagy protein 1 (AMBRA1)	HEK293T cells	TRIM32ΔRING	Autophagy (atrophic muscle)	Not tested	Di Rienzo, et al., 2019
Unc-51 like autophagy activating kinase 1 (ULK1)	HEK293T cells	TRIM32C39S	Autophagy (atrophic muscle)	PolyUb (K63)	Di Rienzo, et al., 2019
PIAS4/PIASy	TRIM32 KO muscle tissue In vitro; Mouse keratinocytes	N/A TRIM32ΔRING	Regeneration (muscle stem cells); Apoptosis (UV-induced cells)	PolyUb PolyUb	Kudryashova, et al., 2012 Albor, et al., 2006
N-myc downstream-regulated gene (NDRG2)	In vitro	N/A	Regeneration (muscle stem cells)	PolyUb	Mokhonova, et al., 2015
c-Myc	C2C12 cells: HEK293T cells	TRIM32C24A; TRIM32C24A	Regeneration (muscle stem cells); Differentiation (neural stem cells)	PolyUb	Nicklas, et al., 2012; Schwamborn et al., 2009
Protein kinase ζ	In vitro; HEK293T cells	TRIM32ΔRING; TRIM32C24A	Differentiation (neural stem cells)	PolyUb	Hillje, et al., 2011
Octamer-binding transcription factor 4 (Oct4)	HEK293T cells	TRIM32ΔRING	Differentiation (iPSCs)	PolyUb	Bahnassawy, et al., 2015
Abl-Interactor 2 (Abi2)	HEK293T cells	TRIM32ΔRING	Tumor suppressor	PolyUb	Kano, et al., 2008
N-myc proto-oncogene protein (MYCN)	In vitro	N/A	Tumor suppressor	PolyUb	Izumi and Kaneko, 2014
Tumor protein 53 (p53)	P53 KO lung cells	TRIM32ΔRING	Tumor suppressor	PolyUb	Liu, et al, 2014
X-linked inhibitor of apoptosis (XIAP)	HEK293T cells	TRIM32ΔRING	Tumor suppressor	PolyUb	Ryu, et al., 2011
AT-Rich Interaction Domain 1A (ARID1A)	HEK293T cells	TRIM32ΔRING	Tumor suppressor	PolyUb	Luo, et al., 2020
OTU domain-containing deubiquitinase with linear linkage specificity (OTULIN)	HEK293T cells	TRIM32ΔRING	Immunity (NFκB activation)	PolyUb (K63)	Zhao et al., 2020
Polymerase basic protein 1 (PB1)	In vitro; TRIM32 KO MEFs	TRIM32C39S	Immunity (viral infection)	PolyUb (K48)	Fu et al., 2015
STING/TMEM173	HEK293T cells	TRIM32ΔRING; TRIM32C39S	Immunity (viral infection)	PolyUb (K63)	Zhang, et al., 2012

[1]tibialis anterior (TA)
